# Pro-Angiogenic Effects of Low Dose Ethoxidine in a Murine Model of Ischemic Hindlimb: Correlation between Ethoxidine Levels and Increased Activation of the Nitric Oxide Pathway

**DOI:** 10.3390/molecules22040627

**Published:** 2017-04-12

**Authors:** Nicolas Clere, Kim Hung Thien To, Samuel Legeay, Samuel Bertrand, Jean Jacques Helesbeux, Olivier Duval, Sébastien Faure

**Affiliations:** 1 MINT, Univ Angers, INSERM, CNRS, Université Bretagne Loire, IBS-CHU, 4 rue Larrey, F-49933 Angers, France; samuel.legeay@univ-angers.fr (S.L.); sebastien.faure@univ-angers.fr (S.F.); 2 Department of Pharmaceutical Pharmacology and Physiology, UFR Santé-School of Pharmacy, University of Angers, F-49045 Angers, France; kht989@mail.missouri.edu; 3 Department of Medical Pharmacology and Physiology, University of Missouri, Columbia, MO 65212, USA; 4 EA 2160, Univ Nantes, Université Bretagne Loire, F-44200 Nantes, France; samuel.bertrand@univ-nantes.fr; 5 School of Pharmaceutical Sciences, University of Geneva, University of Lausanne, Quai Ernest-Ansermet 30, CH-1211 Geneva 4, Switzerland; 6 SONAS, SFR QUASAV 4207, UPRES EA921, Univ Angers, Université Bretagne Loire, F-49035 Angers, France; jean-jacques.helesbeux@univ-angers.fr (J.J.H.); olivier.duval@univ-angers.fr (O.D.)

**Keywords:** ischemia, ethoxidine, nitric oxide, VEGF, angiogenesis, neovascularization

## Abstract

Ethoxidine, a benzo[*c*]phenanthridine derivative, has been identified as a potent inhibitor of topoisomerase I in cancer cell lines. Our group has reported paradoxical properties of ethoxidine in cellular processes leading to angiogenesis on endothelial cells. Because low concentration ethoxidine is able to favor angiogenesis, the present study aimed to investigate the ability of 10^−9^ M ethoxidine to modulate neovascularization in a model of mouse hindlimb ischemia. After inducing unilateral hindlimb ischemia, mice were treated for 21 days with glucose 5% or with ethoxidine, to reach plasma concentrations equivalent to 10^–9^ M. Laser Doppler analysis showed that recovery of blood flow was 1.5 fold higher in ethoxidine-treated mice in comparison with control mice. Furthermore, CD31 staining and angiographic studies confirmed an increase of vascular density in ethoxidine-treated mice. This ethoxidine-induced recovery was associated with an increase of NO production through an enhancement of eNOS phosphorylation on its activator site in skeletal muscle from ischemic hindlimb. Moreover, real-time RT-PCR and western blots have highlighted that ethoxidine has pro-angiogenic properties by inducing a significant enhancement in *vegf* transcripts and VEGF expression, respectively. These findings suggest that ethoxidine could contribute to favor neovascularization after an ischemic injury by promoting the NO pathway and VEGF expression.

## 1. Introduction 

**Figure 1:**
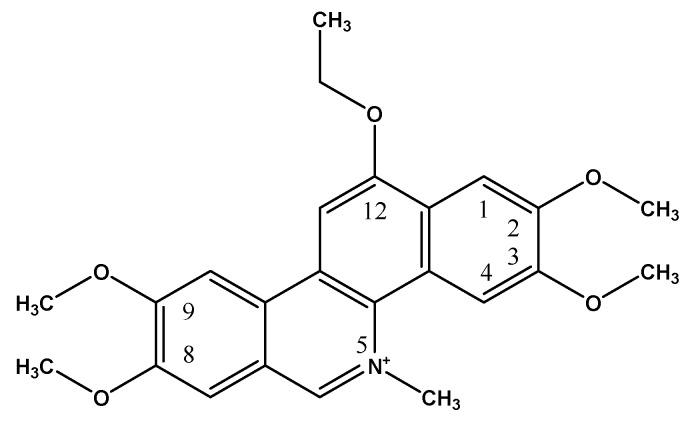
Chemical structure of ethoxidine.

DNA topoisomerases I are essential enzymes that control DNA supercoiling and torsional strain during processes such as replication, transcription and DNA repair [1] whose inhibition is involved in the treatment of many cancers. Ethoxidine, a benzo[*c*]phenanthridine, is a molecule that was synthesized in the late 90s to inhibit DNA topoisomerase I activity (Figure 1) [2]. Although this molecule is not currently used in cancer treatment, many studies have confirmed its anti-proliferative properties on various in vitro models of tumor cells (HT-29, A-549, MCF-7, Caov3) at concentrations between 10^−9^ to 10^−5^ M [3,4]. 

Furthermore, topoisomerase I is highly expressed in endothelial cells [5,6] and various studies have suggested beneficial properties of topoisomerase I inhibitors in the regulation of angiogenesis. For instance, in a model of human umbilical venous endothelial cells (HUVEC), it has been shown that inhibition of topoisomerase I by camptothecin regulates pre-mRNA splicing of endothelial nitric oxide synthase (eNOS), thereby modulating the production of nitric oxide (NO) [7]. In another study performed on HUVEC treated with topoisomerase I inhibitors derived from indenoisoquinoline, antiangiogenic properties have been observed through a significant decrease of vascular endothelial growth factor (VEGF) expression [8]. These findings have been confirmed by in vivo studies in which it has been shown a significant decrease of microvessel growth in mouse cornea model treated with topoisomerase inhibitors [9]. Recently, in clinical samples of various tumors, an association between topoisomerase inhibition and a decrease of VEGF or CD31 expressions has been reported [10]. 

The anti-proliferative properties of various topoisomerase I inhibitors (camptothecin, irinotecan, topotecan, ethoxidine) have been extensively studied in different tumor cell models for concentrations between 10^−9^ and 10^−5^ M [11,12]. However, since no study has evaluated the properties of ethoxidine in the regulation of angiogenesis, our group has showed paradoxical properties of ethoxidine in cellular processes leading to angiogenesis. Thus, in two models of endothelial cells (HUVEC and EaHy.926), it has been shown that low concentration ethoxidine (10^−9^ M) is able to favor angiogenesis through an enhancement of NO production and VEGF expression while a high concentration (10^−5^ M) inhibits angiogenesis by decreasing the expression of pro-angiogenic mediators [13]. Therefore, these findings suggest a dual therapeutic interest of ethoxidine in both treatment of tumor angiogenesis at high concentration and in post-ischemic pathological situations, at low concentration. In this last indication, ethoxidine could promote neovascularization, essential to restore blood flow in ischemic tissues. 

The main objective of this study was to confirm pro-angiogenic properties of ethoxidine in an in vivo model and then, a mouse model of hindlimb ischemia has been used in order to investigate the capacity of low concentration ethoxidine to promote neovascularization process. Interestingly, this study will help to better understand the pro-angiogenic properties of ethoxidine in response to ischemia and is a first step in the development of new therapies for ischemic diseases.

## 2. Results

### 2.1. Ethoxidine Does Not Modify Blood Pressure or Body Weight

At the beginning of the experimental protocol, body weight was equivalent in various groups of mice (21.15 ± 0.39 g for control mice versus 21.74 ± 0.35 g for ethoxidine-treated mice). During the protocol, no weight change has been shown in each group. Thus, after 21 days with ethoxidine, no significant change has been reported between control mice (22.83 ± 0.20 g) and ethoxidine-treated mice (24.13 ± 0.24 g). Furthermore, before the experimental protocol, no difference has been shown in each group of mice (132.67 ± 2.14 mmHg for control mice versus 133.86 ± 2.90 mmHg for mice treated with ethoxidine). After 21 days, ethoxidine had no significant effect on blood pressure (128.60 ± 0.95 mmHg for control mice *versus* 131.71 ± 1.87 mmHg for mice treated with ethoxidine).

### 2.2. Ethoxidine Promotes Blood Flow Recovery after Induction of Hindlimb Ischemia

**Figure 2:**
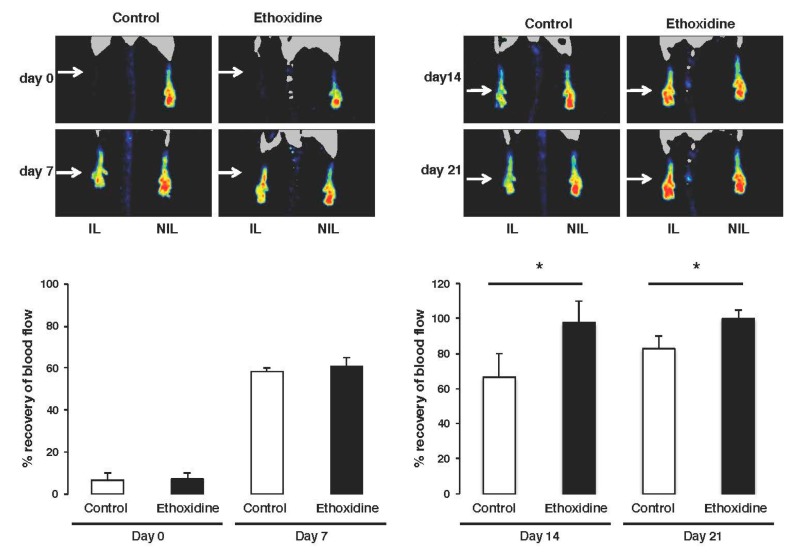
Ethoxidine induced blood flow recovery. Evaluation of neovascularization 7, 14 and 21 days after femoral artery ligation in mice treated with ethoxidine for 21 days. Observations of the recovery of blood flow during the treatment and graphical representation of the percentage recovery over time. Results are means ± SEM as the ischemic/non-ischemic leg ratio (n = 6 mice/group; * *p* < 0.05; IL: ischemic leg; NIL: non-ischemic leg).

The femoral artery ischemia induced a complete loss of skin perfusion on both groups. After ligation, no difference in recovery has been found between control mice (6.61% ± 0.97%) and ethoxidine-treated mice (7.18% ± 1.33%) compared to non-ischemic leg group. The initiation of blood flow recovery in ischemic leg began seven days after ligation both in control group and in the ethoxidine-treated group. After this first week, blood flow is partially restored with rates of 58.34% ± 4.34% and 60.87% ± 5.06% in control mice and ethoxidine-treated mice, respectively. After fourteen days, a significant difference in blood flow recovery has been shown in ethoxidine-treated mice (97.72% ± 6.18%) compared to control mice (66.60% ± 12.30%). At the end of the experimental protocol, a full blood flow recovery has been observed in ethoxidine-treated mice (108.67% ± 9.29%) compared to control mice (82.98% ± 3.24%) (Figure 2).

### 2.3. Ethoxidine Enhances Vascular Density after Induction of Hindlimb Ischemia

**Figure 3:**
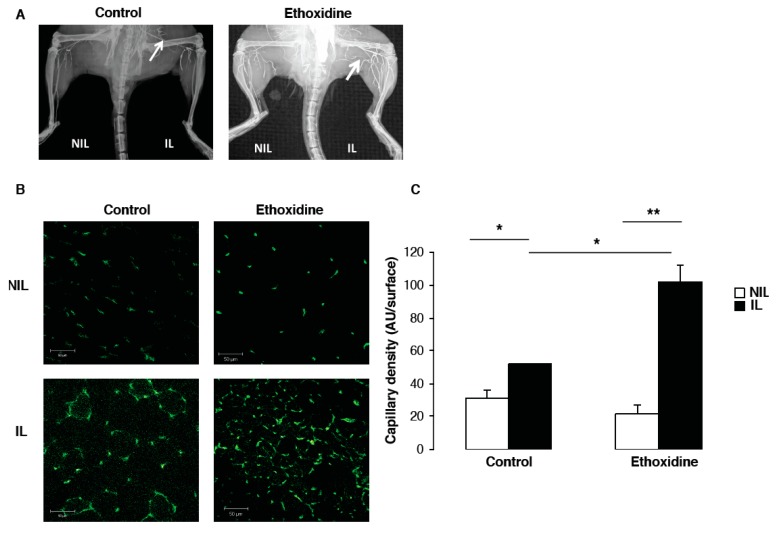
Ethoxidine promotes vascular density in ischemic hindlimb. Vascular density has been evaluated by (**A**) angiography and (**B**) CD31 staining on sections of muscles from mice treated or not with ethoxidine. Capillary density has been quantified in ischemic (IL) or non-ischemic (NIL) leg (n = 6 mice/group; 3 fields have been measured on 3 different sections per muscle; * *p* < 0.05; ** *p* < 0.01).

At the end of the experimental protocol, the analysis of vascular density by angiography revealed the development of a microvascular network in ischemic leg from ethoxidine-treated mice in comparison with control mice. However, no difference has been observed in non-ischemic leg of ethoxidine-treated mice or control mice (Figure 3A).

Data from angiographic analysis have been confirmed by capillary density measurement (Figure 3B). After 21 days, in the control group, a significant increase of vascular density has been shown in ischemic leg (51.77 ± 0.44 units/area) compared to non-ischemic leg (31.04 ± 5.04 units/area). Similarly, in the group of mice treated with ethoxidine, a significant enhancement of capillary density has been observed between ischemic leg (102.08 ± 10.33 units/area) and non-ischemic leg (21.33 ± 5.78 units/area). Interestingly, a significant increase of vascular density has been shown between ischemic leg of ethoxidine-treated mice (102.08 ± 10.33 units/area) and control mice (51.77 ± 0.44 units/area) (Figure 3C).

### 2.4. Ethoxidine Does Not Inhibit Topoisomerase I Activity in Ischemic Hindlimb

**Figure 4:**
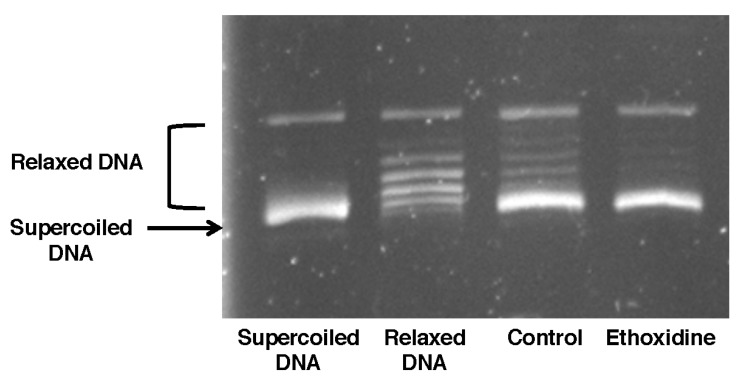
Effects of ethoxidine on topoisomerase I activity in skeletal muscle samples. Tissues have been resected from mice treated or not with ethoxidine for 21 days. Topoisomerase I activity was examined by 1% agarose gel electrophoresis with ethidium bromide and then, was determined by DNA status. Relaxed DNA indicates topoisomerase I activity whereas supercoiled DNA indicates a lack of topoisomerase I activity. No topoisomerase I activity has been highlighted in muscle from both control and ethoxidine-treated mice (results are representative of the analysis performed on 5 mice per group).

The activity of topoisomerase has been studied on skeletal muscle from mice treated or not with ethoxidine. In samples from mice control, DNA was not relaxed and a DNA supercoiled form was observed. Likewise, results showed that ethoxidine was not able to inhibit the changes in the superhelical duplex DNA state suggesting a lack of inhibition of topoisomerase I (Figure 4). 

### 2.5. Ethoxidine Induces NO Production by Enhancing eNOS Activity in Skeletal Muscle

**Figure 5:**
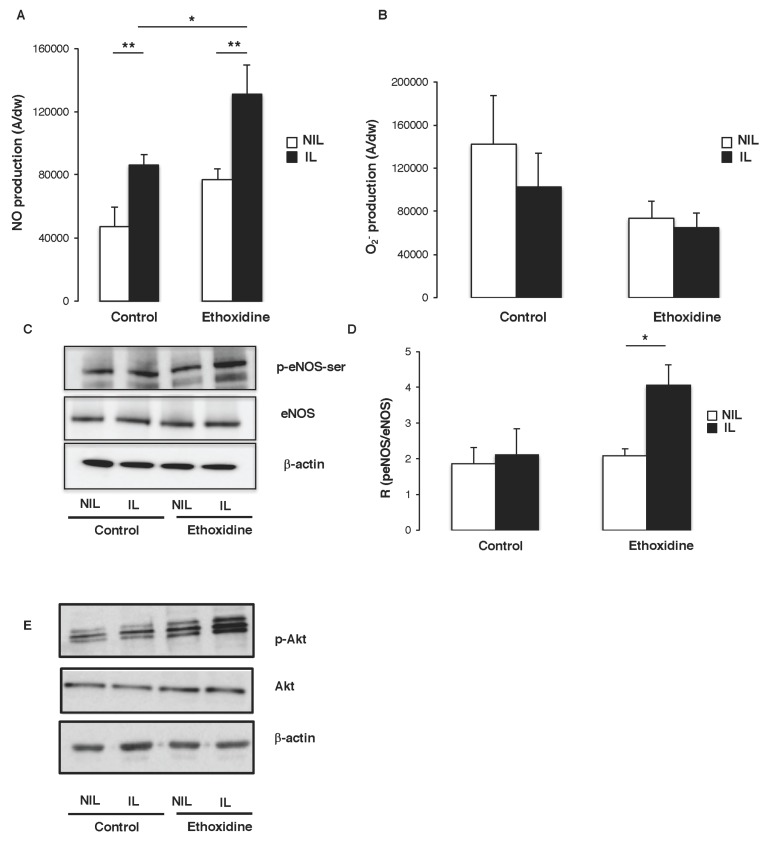
Influence of ethoxidine in the regulation of NO production. Tissues have been resected from mice treated or not with ethoxidine for 21 days and (**A**) quantification of the amplitude of the NO-Fe(DETC)_2_ complex signal. Values are expressed as amplitude/mg of dried weight of skeletal muscle in arbitrary unit. Ischemia is able to enhance NO production and ethoxidine potentiates this increase. Results are means ± SEM (*n* = 6 mice/group; * *p* < 0.05; ** *p* < 0.01; IL: ischemic leg; NIL: non-ischemic leg); (**B**) Quantification of O_2_^−^ production by EPR. Values are expressed as amplitude/mg of dried weight of skeletal muscle in arbitrary unit. Results are means ± SEM (n = 6 mice/group; * *p* < 0.05; ** *p* < 0.01; IL: ischemic leg; NIL: non-ischemic leg); (**C**) Western blot revealed eNOS expression and its phosphorylation on its activator site (Ser-1177); (**D**) Ethoxidine enhances the ratio between p-eNOS-Ser and eNOS in ischemic hindlimb. Results are means ± SEM (n = 6 mice/group; * *p* < 0.05; IL: ischemic leg; NIL: non-ischemic leg). (**E**) Western blots showed Akt expression and phosphorylation. β-actin control was included. Data are representative of four separate blots (IL: ischemic leg; NIL: non-ischemic leg).

At the end of the protocol, in the control group, a significant increase of NO production has been revealed in muscle from ischemic leg (85,993 ± 4639 A/mg) compared to non-ischemic leg (47,308 ± 12,247 A/mg). Furthermore, in the group of mice treated with ethoxidine, a significant increase of NO production has been observed in ischemic leg (131,122 ± 18,545 A/mg) in comparison with non-ischemic leg (76,969 ± 6751 A/mg). Interestingly, a significant enhancement of NO production has been found between ischemic leg of ethoxidine-treated mice (131,122 ± 18,545 A/mg) and control mice (85,993 ± 4639 A/mg) (Figure 5A). Analysis of O_2_^−^ production showed no significant difference between muscle from ischmemic and non-ischemic leg. In ethoxidine-treated mice, no difference of superoxide anions production has been observed (Figure 5B).

To determine the molecular pathways governing the enhancement of NO release induced by ethoxidine, the expression and activation of eNOS were analyzed by Western blot. In control mice, no change in eNOS expression has been reported between ischemic and non-ischemic leg. However, in ethoxidine-treated mice, a significant increase of eNOS expression has been shown in ischemic leg compared to non-ischemic leg. While, in mice control, no changes in eNOS phosphorylation have been observed between ischemic and non-ischemic leg, a significant increase of eNOS phosphorylation has been described in ischemic compared to non-ischemic leg (Figure 5C). These previous data have been confirmed by calculating the ratio between phosphorylated eNOS at its activator site and the total amount of the enzyme. Indeed, in control mice, no change ratio has been reported between ischemic and non-ischemic leg whereas, in ethoxidine-treated mice, a significant increase in the ratio has been shown in ischemic leg in comparison with non-ischemic leg (Figure 5D).

It has been well known that the activation of Akt directly phosphorylates eNOS at its activator site Ser-1177 and subsequently increases NO production. Interestingly, ethoxidine did not alter Akt expression but stimulated its phosphorylation on its activator site (Figure 5E).

### 2.6. Ethoxidine Promotes VEGF Expression in Skeletal Muscle from Ischemic Leg 

**Figure 6:**
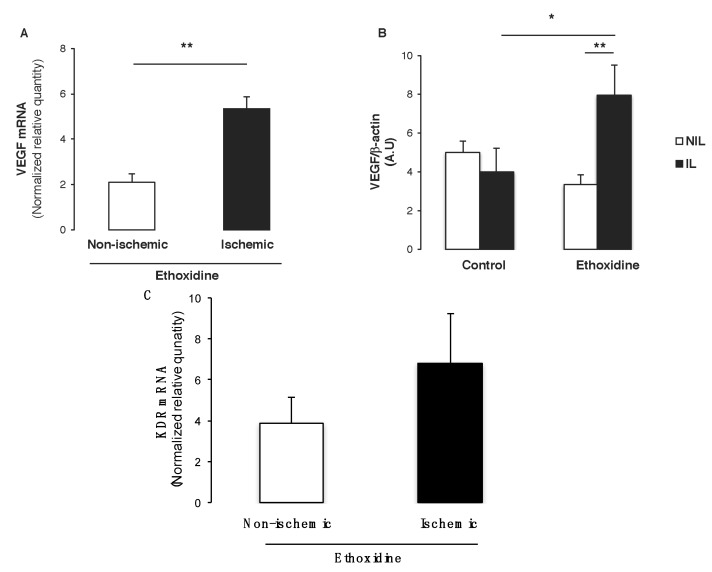
Ethoxidine enhances VEGF expression in skeletal muscle. Tissues have been resected from mice treated or not with ethoxidine for 21 days and (**A**) quantitative RT-PCR analysis was conducted on total RNA from mice skeletal muscle to quantify *vegf* transcripts. Values are expressed as ratio of mRNA expression ethoxidine-treated/control mice from ischemic leg versus non ischemic leg (*n* = 6 mice per groups; ** *p* < 0.01); (**B**) Ratio between VEGF expression and β-actin expression. VEGF expression is enhanced in ischemic leg from ethoxidine-treated mice in comparison with control mice. Results are means ± SEM (n = 6 mice/group; * *p* < 0.05; ** *p* < 0.01; IL: ischemic leg; NIL: non-ischemic leg); (**C**) Quantification of *kdr* transcripts by quantitative RT-PCR. Values are expressed as ratio of mRNA expression ethoxidine-treated/control mice from ischemic leg *versus* non ischemic leg (n = 6 mice per groups).

Since VEGF is the main pro-angiogenic factor involved in neovascularization, both mRNA level and protein expressions have been determined by real-time RT-PCR and western blot, respectively. Interestingly, ethoxidine was able to enhance significantly *vegf* level in ischemic hindlimbs in comparison with non-ischemic hindlimbs (Figure 6A). Furthermore, analysis of VEGF expression by Western blot revealed that in control mice, ischemia did not modify its expression whereas in ethoxidine-treated mice a significant increase of VEGF has been observed in samples from ischemic hindlimbs. Interestingly, in ischemic samples, ethoxidine enhanced significantly VEGF expression (Figure 6B). Finally, analysis of *kdr* mRNA expression (encoding VEGFR2) revealed an enhancement of this gene expression in ischemic samples in comparison with non-ischemic samples (Figure 6C). Moreover, ethoxidine did not modify *flt1* mRNA expression (encoding VEGFR1) either in non-ischemic or in ischemic mice (*data not shown*).

## 3. Material and Methods

### 3.1. Mouse Model of Hindlimb Ischemia

Male C57BL6 mice, 8 weeks of age (Janvier Laboratories, Le Genest-Saint-Isle, France) have been used. Mice were anesthetized with isoflurane and underwent surgery to induce unilateral hindlimb ischemia. The ligation was performed on the left femoral artery proximal to the bifurcation to the saphenous and popliteal arteries [14]. After 21 days, mice were euthanized and tissues were sampled for biochemical and histological analysis. The procedure followed in the care and euthanasia of the study was in accordance with the European Community standards on the care and use of laboratory animals. Furthermore, the ethical committee has approved the present protocol. All animal studies were carried out using approved institutional protocols and were conformed the *Guide for the Care and Use of Laboratory Animals* published by US National Institutes of Health (NIH Publication No. 85–23, revised 1996). According to ethical rules, mice were sacrificed by CO_2_ asphyxia after isoflurane anesthesia. To study the properties of ethoxidine on in vivo neovascularization, two groups of mice with unilateral ischemia has been used: the first group has been treated for 21 days with ethoxidine (0.14 ng/kg/day), corresponding to a plasma concentration of ethoxidine equivalent to 10^−9^ M; the second group (control mice) has been treated for 21 days with glucose 5% alone. Administration of ethoxidine and glucose 5% have been realized using an Alzet^®^ osmotic mini-pump (model 2004, Cupertino, CA, USA).

### 3.2. Blood Pressure Measurement

Non-invasive blood pressure was measured by tail-cuff method (Letica, Barcelona, Spain). Briefly, all vigilant, immobilized and non-anesthetized animals were trained everyday over a period of a week to get accustomed to the device. Measurements were performed 7, 14 or 21 days after surgery. A total of 6 consecutive readings of systolic pressure were daily recorded and averaged.

### 3.3. Evaluation of Plasma Concentration of Ethoxidine

Each sample was prepared in triplicate. 25 µL of internal standard has been added to 1.3 µg/mL acetonitrile (ACN) solution (as internal standard), 12.5 µL of acetonitrile (HPLC grade) and 12.5 µL of serum. Proteins were removed by centrifugation for 2 min at 100% (Mikroliter Zentrifugen, Hettich, Tuttlingen, Germany) and then, the samples have been analysed with LC-MS after a dilution by 4 (40 µL in 160 µL) in water.

Ethoxidine and the internal standard were detected by tandem mass spectrometry in the multiple reaction monitoring (MRM) mode on a Quattro micro™ API mass spectrometer (Waters, Milford, MA, USA) with an electrospray ionization (ESI) interface coupled with Agilent LC system (Hewlett Packard, Ramsey, NJ, USA). The ESI conditions were as follows: capillary voltage 2500 V; cone voltage 40 V; extractor 2 V; RF Lens 0.5 V; source temperature 150 °C; desolvation temperature 350 °C; cone gas flow 30 L h^−1^ and desolvation gas flow 900 L h^−1^. Detection was performed in the positive ion mode. The MRM transitions are: ethoxidine 408 → 364 and JJ6 472 → 378 (parent > daughter). The separation was carried out on a Kinetex C18 column (2.5 µm, 100 A, 100 × 3.0 mm, Phenomenex, Torrance, CA, USA). A gradient was performed at 35 °C, at a flow rate of 0.45 mL min^−1^ with the following solvent system: A = 0.1% formic acid/water, B = 0.1% formic acid/ACN. A gradient starting from 5% B to 95% B in 20 min followed by 10 min cleaning at 95% B and a 10 min equilibration time at 5% B, was used for the analysis. The analysis was performed injecting 10 µL of sample in triplicate (this leads to nine replicates for one serum sample). 

### 3.4. DNA Topoisomerase I Activity Assays

Measurement of the DNA topoisomerase I assay has been realized on skeletal muscle from both control and ethoxidine-treated mice. Topoisomerase I activity has been determined by using a corresponding kit (TopoGEN, Inc., Buena Vista, CO, USA) following the manufacturer’s instructions. Briefly, samples have been incubated with supercoiled DNA in 1X reaction buffer for 30 min at 37 °C and terminated by adding stop leading buffer. Then, samples have been loaded on a 1% agarose gel containing ethidium bromide and have been run in 1X TAE buffer. Gels have been photographed using a UV transilluminator and relaxation of supercoiled DNA has been subsequently assessed.

### 3.5. Laser-Doppler Whole Body Imaging

To determine the evolution of ischemia, after 0, 7, 14 and 21 days of ligation, laser Doppler perfusion was performed in anesthetized mice. Animals were settled in an incubator (MMS, Chelles, France) that allowed maintenance of a stable cutaneous temperature (35.0 ± 0.5 °C) throughout the experiment. Perfusion was then measured in the feet using a laser Doppler flow probe (PF 408; Perimed, Craponne, France). Blood flow was recorded for 5 min. At least two flow measurements were performed at each time point at 0, 7, 14 and 21 days. Blood flow perfusion was expressed as a ratio of left (ischemic) to right (non-ischemic) leg. 

### 3.6. Angiography

Arterial density was evaluated by high-definition angiography 21 days after ligation. Briefly, mice were anesthetized (sodium pentobarbital, 50 mg/kg, *i.p.*) and a contrast medium (barium sulfate, 6 g/mL) was injected through a catheter introduced into the abdominal aorta. Two images were acquired per animal using a digital X-ray transducer (Faxitron X-Ray Corporation, Wheeling, IL, USA).

### 3.7. Arteriolar and Capillary Density

Assessing capillary and arteriolar densities has completed angiographic analysis. Briefly, ischemic and non-ischemic muscles were dissected and progressively frozen in isopentane solution cooled in liquid nitrogen. Sections (7 μm) were first incubated for 30 min in PBS containing 5% BSA at room temperature, then for 1 h with rabbit polyclonal antibody directed against total PECAM (BD Bioscience, San Jose, CA, USA) to identify capillaries. Capillary densities have been calculated in three randomly chosen fields of a definite area for each muscle section.

### 3.8. NO Determination by Electron Paramagnetic Resonance (EPR)

Detection of NO production was performed using Fe^2+^ diethyldithiocarbamate (DETC, Sigma-Aldrich, St. Louis, MO, USA) as spin trap. Briefly, skeletal muscle was obtained from mice treated or not by ethoxidine for 21 days. Tissue was dissected and incubated for 30 min in Krebs-HEPES buffer containing CaCl_2_ (3 mM) and L-arginine (0.8 mM) to assess NO production. A diethyldithio-carbamate–iron(II) complex (Fe[DETC]_2_) solution was added to organ and incubated for 45 min at 37 °C. Skeletal muscle was immediately frozen using liquid nitrogen. NO measurement was performed on a table-top x-band spectrometer Miniscope (Magnettech, MS200, Berlin, Germany) as previously described. Values are expressed in unit/mg weight of dried tissue. For O_2_^−^ quantification, skeletal muscles were allowed to equilibrate in deferoxamine-chelated Krebs-Hepes solution containing 1-hydroxy-3-methoxycarbonyl-2,2,5,5-tetramethylpyrrolidin (CMH; Noxygen, Mainz, Germany) (500 µmol/L), deferoxamine (25 µmol/L), and DETC (5 µmol/L) under constant temperature (37 °C) for 20 min. Cells were then scrapped and frozen in plastic tubes and analyzed by EPR spectroscopy. Values are expressed as units/mg weight of dried tissue.

### 3.9. Quantitative Real-Time Reverse Transcription–Polymerase Chain Reaction Analysis 

Skeletal muscle from both ischemic and non-ischemic legs were frozen and used to investigate mRNA levels of VEGF transcripts by real-time reverse transcription–polymerase chain reaction (RT–PCR). RT–PCR analyses were carried out by Plateforme d’Analyse Cellulaire et Moléculaire (PACEM) from Angers University on Chromo 4™ (Bio-Rad, Hercules, CA, USA) using the SYBR Green PCR Master Mix (Invitrogen, Carlsbad, CA, USA). Quantifications were realized according to the ΔCt method and the relative gene expression were normalized using the geometric mean of three housekeeping genes. Data were expressed as a ratio of ischemic on non-ischemic gene-level expression.

### 3.10. Western Blot

Skeletal muscle from both ischemic and non-ischemic legs was removed and frozen. Samples were collected and homogenized (Polytron Pro 250, Bioblock Scientific, Illkirch, France). Proteins were separated by SDS-PAGE. After migration, proteins were transferred to PVDF blotting membranes (Immobilon-P, Millipore, Billerica, MA, USA). These were incubated overnight 4 °C with eNOS (BD Bioscience), p-eNOS Ser1177, pAkt, Akt (Cell Signaling, Beverly, MA, USA). Protein expression has been normalized with β-actin (Sigma Aldrich, Saint-Louis, MO, USA). Membranes were analyzed using the ECL-Plus Chemiluminescence kit (Amersham Biosciences, Piscataway, NJ, USA).

### 3.11. Statistical Analysis

Data are expressed as mean ± SEM, *n* represents the number of mice used in this protocol. Statistical analyses were performed by ANOVA followed by Bonferroni’s test. *p* < 0.05 was considered to be statistically significant.

## 4. Discussion

The present study provides evidence that low concentration ethoxidine is able to promote both neovascularization in post-ischemic context and the resultant blood flow recovery in a murine model of hindlimb ischemia. To explain these properties, this study suggests that low concentration ethoxidine (10^−9^ M) involves NO pathway and promotes VEGF gene and protein expressions. Taken together, these data highlight pro-angiogenic properties of low dose ethoxidine associated with the formation of new blood capillaries, an important component of pathological tissue repair in response to ischemia. 

Only one study has investigated the metabolism of ethoxidine in human and rodent cell models. Thus, for first-pass effect, it has been reported that metabolism of ethoxidine induces the formation of different metabolites [15]. However, in order to overcome the possible effects of these metabolites, and to evaluate the specific properties of ethoxidine, the subcutaneous route has been chosen to deliver this drug. Ethoxidine has been administrated through osmotic mini-pumps during the protocol and the analysis of plasma concentration confirmed an average plasma concentration between 18.34 and 46.97 nM ethoxidine (*data not shown*). The half maximal inhibitory concentration (IC_50_) of topoisomerase has been determined in a previous study in which it has been reported an IC_50_ of about 0.22 μM for ethoxidine [4]. This last concentration is much higher than that used in our study and this could justify the absence of inhibition of topoisomerase activity described in the present work. Under our experimental conditions, our present findings suggest that ethoxidine is able to enhance neovascularization through a mechanism independent of topoisomerase inhibition. The absence of topoisomerase inhibition may be beneficial for a therapeutic use of ethoxidine, since topoisomerase inhibitors have numerous fatal side effects, as previously described with irinotecan [16,17]. Our findings suggest that low dose ethoxidine may prevent toxic side effects and these properties would ensure a better risk-benefit ratio for this molecule in the treatment of ischemic diseases.

This study of post-ischemic neovascularization has been performed on an in vivo model of C57/BL6 mice during 21 days as previously described by Madeddu et al. [18]. This in vivo model is appropriate to study ischemic angiogenesis, a major physiological process described in cardiac or cerebral ischemic diseases. In control mice, we showed that ischemic-induced angiogenesis has been associated with an enhancement of NO, eNOS and VEGF expression, as it has been described in previous studies conducted on ischemic rat model [19,20]. Furthermore, we found that the blood flow recovery has been initiated from the seventh day after the initiation of treatment with ethoxidine, to reach a maximum value at the end of the protocol. These findings are consistent with previous works performed on the same murine model of femoral artery ligation [21] in which both the initiation of blood flow recovery and a maximum value have been observed one and four weeks after the ligation, respectively. Despite these latest results, no change in blood flow has been observed in non-ischemic legs from ethoxidine-treated mice. These observations suggest that ischemia is a prerequisite for potentiating in vivo pro-angiogenic properties of ethoxidine as previously described by Baron-Menguy et al. on post-ischemic neovascularization models induced by low doses red wine polyphenols (0.2 mg/kg) [22]. 

The formation of new blood capillaries is an important component of pathological tissue repair in response to ischemia. The development of an effective collateralization in the ischemic area involves angiogenesis, which is the sprouting of new capillaries, and arteriogenesis, defined as the development of arterial structures from small preexisting collateral vessels [23]. The present study shows that, after 21 days, low dose ethoxidine is able to increase significantly vascular density suggesting that ethoxidine modulates preferentially angiogenesis rather than arteriogenesis. In fact, in the present study, we found that CD31 staining is significantly increased in ischemic leg treated with ethoxidine. Because VEGF has been described as a major mediator in the regulation of angiogenesis rather than arteriogenesis [24,25], analysis of VEGF expression has been considered by real-time RT-PCR and western blot. Thus, the present study describes a significant increase of both VEGF mRNA level and its protein expression. Although we have not evaluated the basal VEGF expression, various studies confirm that under normal conditions (before ischemia), the basal rate of VEGF is very low and slightly different than control conditions used in the present work. Then, in a model of unilateral pulmonary arterial ischemia performed on five-week old C57/BL6 mice, no significant difference on VEGF mRNA or protein baseline expression between control and sham mice has been reported [26]. Furthermore, in another ischemic model conducted on C57/BL6 mice [27], a significantly lower baseline level of VEGF has been detected before inducing ischemia. Although Flt1/VEGFR-1 has a high affinity for VEGF (Kd = 1~10 pM), which is one order higher than that of KDR/VEGFR-2, its tyrosine kinase activity is approximately 10-fold weaker than that of KDR/VEGFR-2. Thus, the major pro-angiogenic signal is generated from the VEGF-activated KDR/VEGFR-2 [28]. Thus, the enhancement of *kdr* transcripts expression suggested pro-angiogenic properties of VEGF mediated by this receptor. As previously described on endothelial cells [13], our data confirmed the involvement of the VEGF pathway in the pro-angiogenic properties of ethoxidine.

The role of NO in the regulation of angiogenesis is well described [29], particularly in the regulation of VEGF expression [30]. Furthermore, it has been reported that ischemia is able to enhance NO production, essential to favor angiogenesis [31]. While these last findings have been confirmed in our study model, the present study reported that, in ischemic conditions, ethoxidine is able to enhance NO production. These data suggest that the pro-angiogenic properties of ethoxidine could be explained by an increase of both production and biological activities of NO, as previously described in in vitro study [13]. Then, from RPE analysis, we reported that in the absence of treatment, ischemia was able to induce NO production without altering the superoxide anion production after 21 days. Moreover, in ischemic situation, ethoxidine increased significantly NO production in comparison with control mice, while reducing superoxide anions production, thereby increasing the biological activities of NO (Figure 4B). Furthermore, several studies, conducted in a model of femoral artery ligation, have confirmed that NO production is secondary to the activation of endothelial NO synthase [32,33]. Similar effects have been observed in the present study in which it has been found that ethoxidine activates NO pathway through a potentiation of eNOS phosphorylation. 

To understand the mechanisms of action by which ethoxidine increases NO production, an analysis of Akt expression and its phosphorylation has been performed by Western Blot. Indeed, in HUVEC model, it has been reported that NO production was mediated by an activation of Akt and then a phosphorylation of eNOS on its activator site (Ser-1177) [34]. No change in Akt expression has been induced by both ethoxidine and ischemia. However, on muscle from ischemic leg from mice treated with ethoxidine, a significant increase of Akt phosphorylation has been observed (*data not shown*). The serine/threonine protein kinase Akt is a downstream effector of PI3K that is activated by a variety of growth factors including those known to induce angiogenesis, such as VEGF and bFGF in endothelial cells [35], suggesting a possible involvement of the PI3K-Akt pathway in the angiogenic process [36]. Our present study suggested a direct mechanism of action for ethoxidine. Indeed, this molecule was able to activate Akt then eNOS to stimulate NO production that is necessary to promote neovascularization.

The results of this study confirm the pro-angiogenic ability of ethoxidine, which is able to induce neovascularization through the activation of NO pathway. Therefore, ethoxidine treatment may significantly contribute to stimulate neovascularization following ischemic injury. Furthermore, in these experimental conditions, the lack of inhibition of topoisomerase activity is an advantage to limit cellular toxic effects involving, in particular, the regulation of gene transcription by topoisomerases. Interestingly, this study confirms that low concentration ethoxidine has no potentially serious side effects and could offer important therapeutic perspectives for the treatment and prevention of ischemic diseases.

## 5. Conclusions

The present study highlights a beneficial effect of ethoxidine in an in vivo model of angiogenesis triggered by ischemia. Interestingly, while ethoxidine, a topoisomerase inhibitor, could be used as antitumor molecule, its low dose in nanomolar range (10^−9^ M) offers new important therapeutic outcomes for the treatment and prevention of ischemic diseases. Furthermore, since effective at low dose, the treatment with 10^−9^ M ethoxidine has potentially no serious adverse effects, a high degree tolerability in addition to a good safety profile.
